# *Wuchereria bancrofti* filaria activates human dendritic cells and polarizes T helper 1 and regulatory T cells via toll-like receptor 4

**DOI:** 10.1038/s42003-019-0392-8

**Published:** 2019-05-07

**Authors:** Suprabhat Mukherjee, Anupama Karnam, Mrinmoy Das, Santi P. Sinha Babu, Jagadeesh Bayry

**Affiliations:** 1 Department of Zoology (Centre for Advanced Studies), Visva-Bharati University, Santiniketan, 731235 India; 2 Institut National de la Santé et de la Recherche Médicale; Centre de Recherche des Cordeliers, Equipe—Immunopathologie et immuno-intervention thérapeutique, Sorbonne Universités, F-75006 Paris, France; 3 Université Paris Descartes, Sorbonne Paris Cité, F-75006 Paris, France; 4 Present Address: Department of Animal Science, Kazi Nazrul University, Asansol, West Bengal 713 340 India

**Keywords:** Immunology, Adaptive immunity, Infection, Innate immune cells, Lymphocytes

## Abstract

Interaction between innate immune cells and parasite plays a key role in the immunopathogenesis of lymphatic filariasis. Despite being professional antigen presenting cells critical for the pathogen recognition, processing and presenting the antigens for mounting T cell responses, the dendritic cell response and its role in initiating CD4^+^ T cell response to filaria, in particular *Wuchereria bancrofti*, the most prevalent microfilaria is still not clear. Herein, we demonstrate that a 70 kDa phosphorylcholine-binding *W. bancrofti* sheath antigen induces human dendritic cell maturation and secretion of several pro-inflammatory cytokines. Further, microfilarial sheath antigen-stimulated dendritic cells drive predominantly Th1 and regulatory T cell responses while Th17 and Th2 responses are marginal. Mechanistically, sheath antigen-induced dendritic cell maturation, and Th1 and regulatory T cell responses are mediated via toll-like receptor 4 signaling. Our data suggest that *W. bancrofti* sheath antigen exploits dendritic cells to mediate distinct CD4^+^ T cell responses and immunopathogenesis of lymphatic filariasis.

## Abstract (web summary)

Mukherjee, Karnam, Das et al. show that *Wuchereria bancrofti* microfilarial sheath antigen induces maturation of human dendritic cells, driving Th1 and regulatory T cell responses. This study illustrates how the most prevalent microfilaria *W. bancrofti* exploits the host innate immunity to cause the immunopathogenesis of lymphatic filariasis.

## Introduction

Lymphatic filariasis is one of the debilitating, disfiguring vector-borne diseases belonging to “neglected tropical diseases” affecting more than 120 million people worldwide^1^. Lymphatic filariasis is caused by microfilariae of *Wuchereria bancrofti* and two species of *Brugia* (*B. malayi* and *B. timori)* that circulate in the blood during night. Among these nematodes, *W. bancrofti* is the principal causative parasite of lymphatic filariasis in human accounting for nearly 90% of infections with lymphedema, lymphangitis, and elephantiasis as major pathological outcomes.

Immunopathological alterations in lymphatic filariasis are mainly caused by multiple facets of host-parasite interactions involving different immune cells (monocytes/macrophages, dendritic cells, granulocytes) and various stages of the filarial parasite (microfilaria, infective larvae and adult)^2^. In general, Th2 cytokines are critical for protection against filarial infection while anti-inflammatory cytokines including IL-10 protect from severe pathology^2^. On the other hand, sustained pro-inflammatory cytokines secreted by innate cells and Th1, Th17 effector cells contribute to immune-mediated pathology^3^. Regulatory T cells, though reduce the inflammatory responses and immunopathologies due to their suppressive functions on effector T cells as well as innate cells^4–6^ and promote basophil activation to induce IL-4 to sustain Th2 responses^7,8^, regulatory T cells also promote survival of parasite and establishment of chronic, asymptomatic infection. Thus, cross-talk between filaria and antigen presenting cells and subsequent CD4^+^ T cell polarization dictates final outcome of filarial infection.

Dendritic cells are professional antigen presenting cells and sentinels of the immune system. They are the key innate cells for mounting adaptive immune response to the pathogens. Dendritic cells uptake the pathogens, process and present the antigens in the context of MHC class II to CD4^+^ T cells^9,10^. By virtue of high expression of co-stimulatory molecules and ability to secrete a wide-range of cytokines, dendritic cells polarize distinct CD4^+^ T responses i.e., Th1, Th2, Th17, and regulatory T cells.

The available reports on cross-talk between filaria and dendritic cells are focused mainly on the laboratory-adapted zoophilic strain *B. malayi*^11,12^. However, despite responsible for 90% of the filaria infection in population, the interaction of *W. bancrofti* with dendritic cells and subsequent CD4^+^ T cell responses remain unexplored. Sheath antigen (~70 kDa) is an immunodominant antigen of *W. bancrofti* and is critical for inflammatory pathology associated with lymphatic filariasis^13^. Our previous investigation has revealed that microfilarial sheath antigen acts as a ligand for Toll-Like Receptor 4 (TLR4) and induces inflammation in macrophages through NF-κB activation^13^. Intriguingly, antibody-mediated blockade of this protein abrogated filarial parasite-induced inflammatory responses in macrophages^13^. In addition to microfilariae, sheath antigen is also present in adult filarid and responsible for the inflammatory consequences induced by the adult stage parasites^14^. Therefore, in view of prime role of dendritic cells in the orchestration of immune response, we investigated the interaction of *W. bancrofti* sheath antigen and dendritic cells.

We demonstrate that *W. bancrofti* sheath antigen, a phosphorylcholine-binding antigen induces maturation of human dendritic cells and secretion of various pro-inflammatory cytokines via TLR4-dependent pathway. Further, analyses of CD4^+^ T cell responses mediated by microfilarial sheath antigen-stimulated dendritic cells revealed that sheath antigen drives predominantly Th1 and regulatory T cell responses. Our data indicate that *W. bancrofti* sheath antigen exploits dendritic cells to mediate CD4^+^ T cell responses and immunopathogenesis of lymphatic filariasis.

## Results

### *W. bancrofti* sheath antigen induces maturation and activation of human dendritic cells

We first explored the outcome of interaction of *W. bancrofti* sheath antigen with dendritic cells on the phenotype. Dendritic cells were differentiated from peripheral blood monocytes of healthy donors of a non-endemic country (France). Our previous report has shown that microfilarial sheath antigen induces proinflammatory responses in macrophages^13^. Based on this previous study, initial experiments were performed with three concentrations (5, 10 and 25 μg) of microfilarial sheath antigen and found that even at 5μg concentration, sheath antigen could induce maturation-associated markers on dendritic cells and was used for all subsequent experiments.

**Fig. 1:**
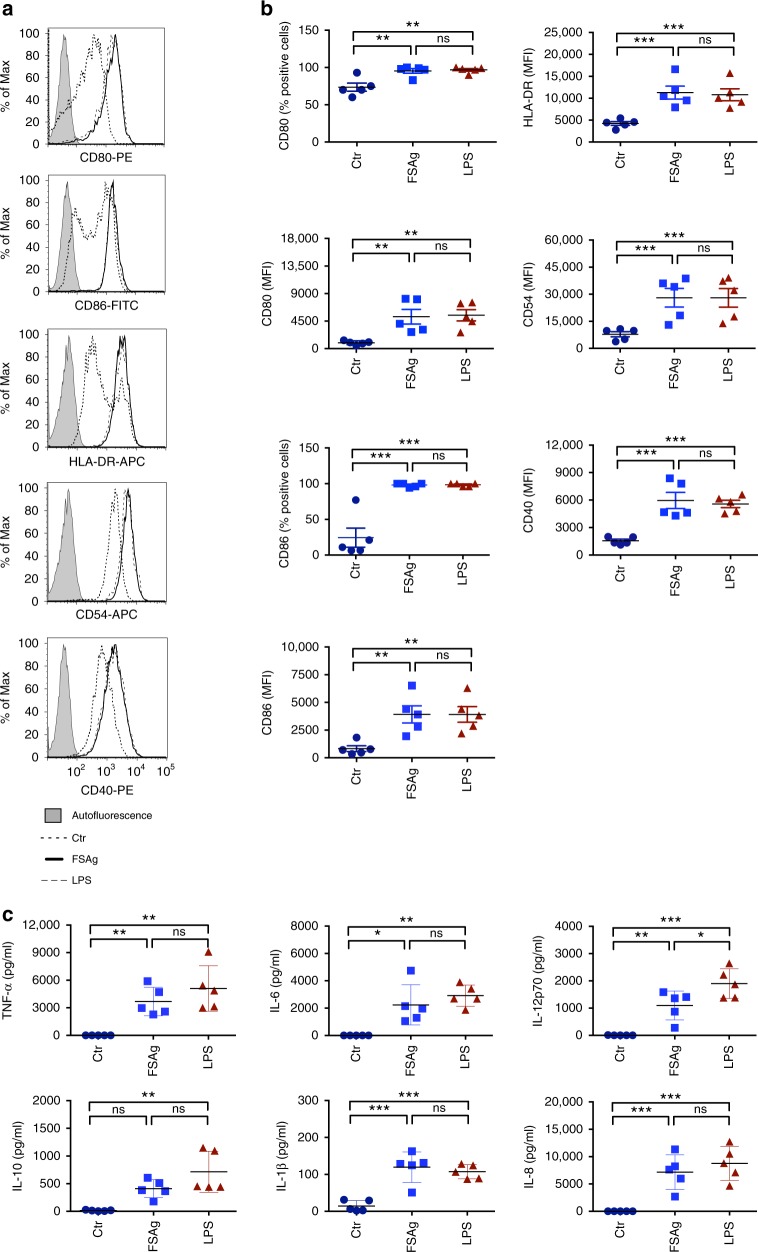
*W. bancrofti* sheath antigen induces maturation and activation of human dendritic cells. **a**–**b** Expression of dendritic cell-maturation markers upon stimulation of cells with microfilarial sheath antigen (FSAg). LPS was used as a positive control. Representative histograms and mean ± SEM (*n* = 5 donors) values of expression (% positive cells or median fluorescence intensities, MFI) of CD80, CD86, HLA-DR, CD54, and CD40. **c** Amount of secretion (mean ± SEM, *n* = 5) of TNF-α, IL-6, IL-12p70, IL-10, IL-1β, and IL-8 (all in pg/ml) by FSAg-stimulated dendritic cells. *P < 0.05; **P < 0.01; ***P < 0.001; ns, not significant as analyzed by one-way ANOVA test. Abbreviation: Ctr, control

Microfilarial sheath antigen induced maturation of dendritic cells evidenced by enhancement in the expression of co-stimulatory (CD80, CD86, CD40), adhesion (CD54) and antigen-presenting (HLA-DR) molecules (Fig. 1a, b). The extent of induction of maturation by microfilarial sheath antigen was similar to lipopolysaccharide (LPS) from *Escherichia coli*, used as a positive control.

Next, we investigated whether induction of maturation of dendritic cells by microfilarial sheath antigen also leads to their activation. Dendritic cells secrete a range of cytokines upon activation that regulate inflammation and CD4^+^ T cell polarization. Microfilarial sheath antigen induced large amounts of TNF-α, IL-6, IL-12p70, and IL-8 (Fig. 1c). Though induced, IL-1β quantity was marginal. However, in contrast to LPS, IL-10 induction by sheath antigen was minimal.

**Fig. 2:**
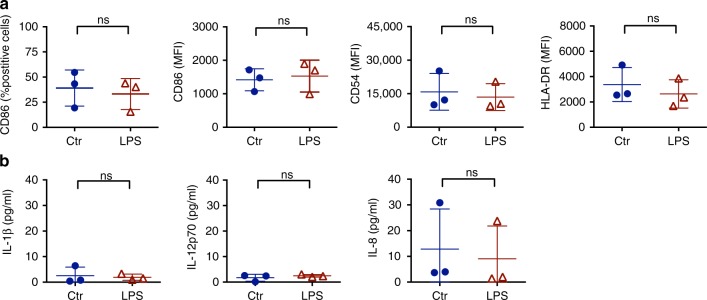
Dendritic cell stimulatory capacity of LPS at a dose equivalent of residual endotoxins in microfilarial sheath antigen. **a** Expression of dendritic cell maturation markers and **b** amount of secretion cytokines (mean ± SEM, *n* = 3, all in pg/ml) upon stimulation of cells with LPS (50 pg/0.5 × 10^6^/ml). ns, not significant as analyzed by two-way Mann–Whitney *U* test. Abbreviation: Ctr, control

We established that the ability of microfilarial sheath antigen to induce dendritic cell maturation was not due to endotoxin/LPS contamination. Endotoxin-free solutions were used for the extraction of sheath antigen and the protein was passed through polymyxin B agarose column before used for the experiments. The endotoxin level in the sheath antigen was 0.46 EU/5 μg of antigen. In order to confirm that dendritic cell stimulatory ability of microfilarial sheath antigen was not due to this residual minute amount of endotoxin in the antigen, we have stimulated dendritic cells with 50 pg/ml of LPS. We did not observe considerable changes in the expression of dendritic cell maturation markers and secretion of inflammatory cytokines at this dose of LPS. The phenotype of dendritic cells represented by CD86, HLA-DR and CD54, and amount of secretion of IL-1β, IL-12p70, and IL-8 by them upon stimulation with 50 pg of LPS were on par with unstimulated cells (Fig. 2a, b).

### *W. bancrofti* sheath antigen induces dendritic cell activation through TLR4

**Fig. 3:**
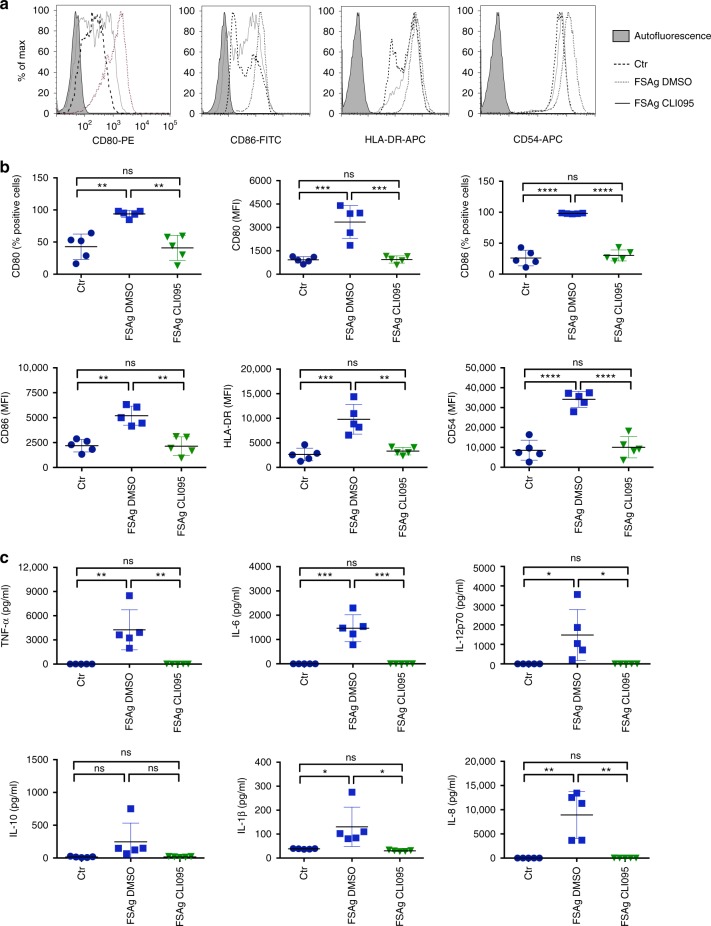
TLR-4 signaling is critical for the induction of dendritic cell activation by *W. bancrofti* sheath antigen. **a**–**b** Effect of CLI095 (TLR4 pathway inhibitor) or dimethyl sulfoxide (DMSO, solvent control) on the expression of microfilarial sheath antigen (FSAg)-induced dendritic cell maturation markers. Representative histograms and mean ± SEM (*n* = 5 donors) values of CD80, CD86, HLA-DR, and CD54 are presented. **c** Inhibitory effect of CLI095 on the secretion (mean ± SEM, *n* = 5) of TNF-α, IL-6, IL-12p70, IL-10, IL-1β, and IL-8 (all in pg/ml) by FSAg-stimulated dendritic cells. *P < 0.05; **P < 0.01; ***P < 0.001; ****P < 0.0001; ns, not significant as analyzed by one-way ANOVA test. Abbreviation: Ctr, control

Our earlier report demonstrated that *W. bancrofti* sheath antigen induces activation of macrophages through TLR4 pathway^13^. Therefore, to investigate whether microfilarial sheath antigen-induced dendritic cell activation was mediated via TLR4, we employed CLI095, a TLR4 signaling inhibitor. As presented in Fig. 3a–c, pre-treatment of dendritic cells with CLI095 completely abolished the stimulatory activity of sheath antigen on the maturation (phenotype) and activation (cytokine secretion) of dendritic cells. These data thus clearly revealed that dendritic cell maturation and activation in response to microfilarial sheath antigen is TLR4-dependent.

**Fig. 4:**
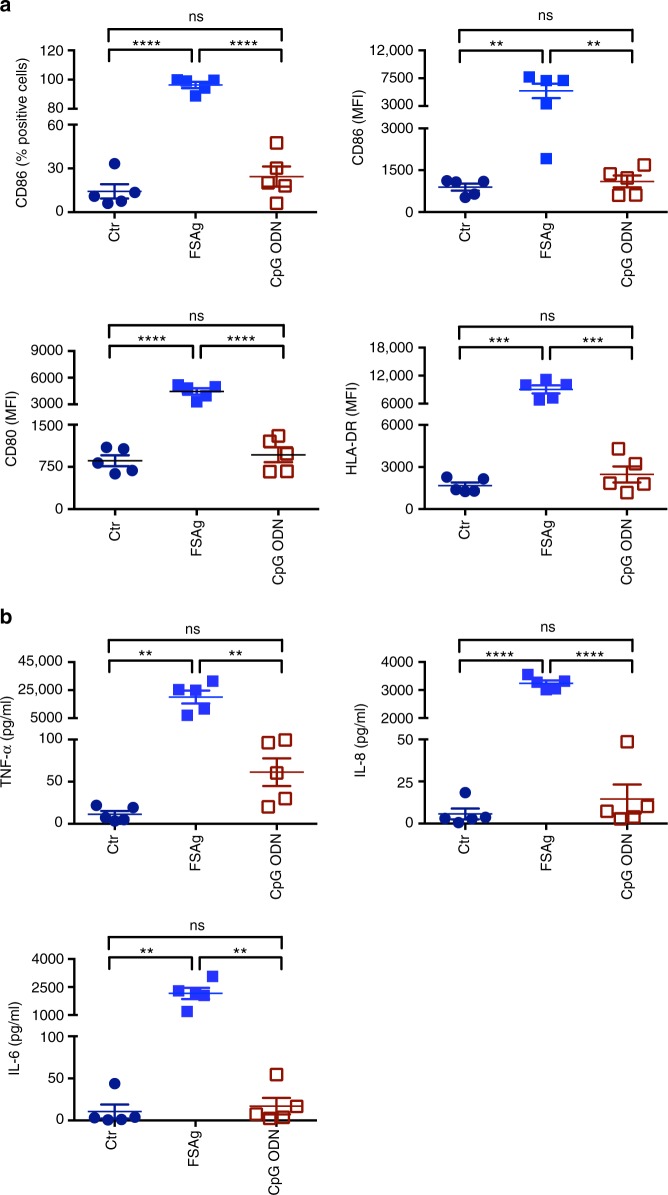
Comparison of dendritic cell stimulatory capacity of *W. bancrofti* sheath antigen and CpG ODN 2006. **a** Expression of dendritic cell-maturation markers CD86, CD80 and HLA-DR (mean±SEM; % positive cells or median fluorescence intensities, MFI) upon stimulation of cells with microfilarial sheath antigen (FSAg) or CpG ODN. Data are from five independent donors. **b** Amount of secretion (mean±SEM, *n* = 5) of dendritic cell cytokines TNF-α, IL-8, and IL-6 (all in pg/ml) under above stimulatory conditions. **P < 0.01; ***P < 0.001; ****P < 0.0001; ns, not significant as analyzed by one-way ANOVA test. Abbreviation: Ctr, control

In view of dependency on TLR4 signaling for the induction of dendritic cell activation by microfilarial sheath antigen, we have compared the stimulatory ability of this antigen with a non-TLR4 agonist by using CpG ODN 2006 (TLR9 agonist) as a control. As shown in Fig. 4a, b, microfilarial sheath antigen is far superior to a TLR9 agonist in its capacity to induce dendritic cell maturation markers and cytokines.

### *W. bancrofti* sheath antigen binds to TLR4 of dendritic cells

**Fig. 5:**
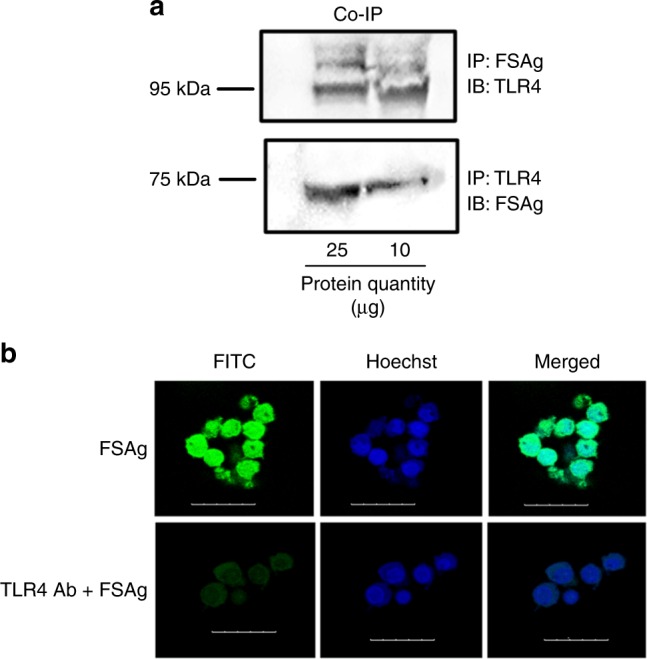
*W. bancrofti* sheath antigen recognizes TLR-4 on dendritic cells. **a** Co-immunoprecipitation of microfilarial sheath antigen (FSAg)–TLR4 complex followed by reciprocal immunoblotting using anti-FSAg or anti-TLR4 antibodies. **b** Binding of FITC-labelled FSAg with dendritic cell-TLR4. Dendritic cells were incubated with FITC-conjugated FSAg. In a separate set of experiments, dendritic cells were pre-treated with anti-TLR4 antibodies followed by incubation with FITC-conjugated FSAg (TLR4 Ab + FSAg). Cells were analyzed by confocal microscopy (scale bar = 25 μm). Nuclei were stained with Hoechst staining. Abbreviations: Ctr, control; IP, Immunoprecipitation; IB, Immunoblot

In view of critical role of TLR4 in mediating microfilarial sheath antigen-induced dendritic cell activation, we asked whether sheath antigen could physically interact with TLR4 on dendritic cells to induce such responses. We performed co-immunoprecipitation of microfilarial sheath antigen–TLR4 complex from dendritic cells either with anti-sheath antigen antibodies or anti-TLR4 antibodies followed by reciprocal immunoblotting with respective antibodies. The immunoblot revealed a clear interaction between microfilarial sheath antigen and TLR4 (Fig. 5a). This physical interaction was further confirmed by immunofluorescence analyses using FITC-labelled sheath antigen wherein it bind dendritic cells by TLR-4-dependent manner as blocking antibodies to TLR4 abrogated sheath antigen binding (Fig. 5b). Thus, these data indicated that microfilarial sheath antigen signals dendritic cell activation by binding to TLR4.

### *W. bancrofti* sheath antigen matured dendritic cells induce predominantly Th1 and regulatory T cell responses

**Fig. 6:**
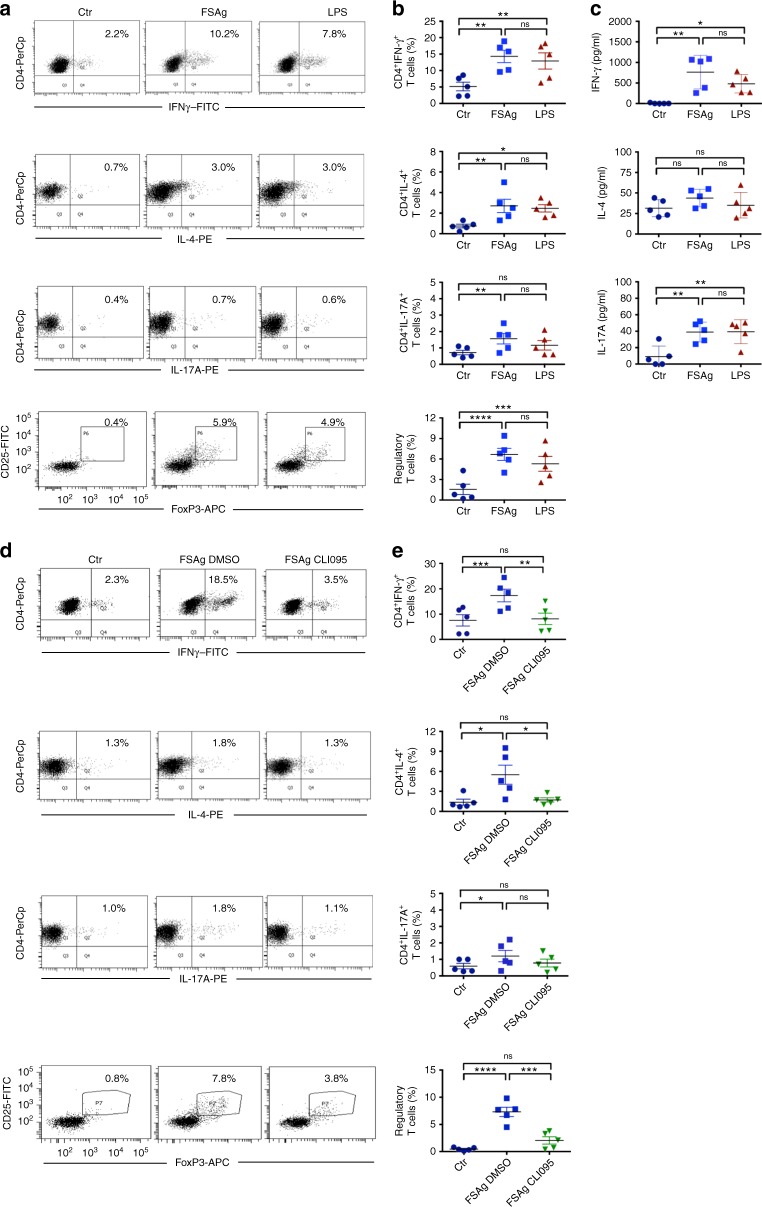
*W. bancrofti* sheath antigen-stimulated dendritic cells induce predominantly Th1 and regulatory T cell responses by TLR4-dependent mechanism. **a**–**b** *W. bancrofti* sheath antigen (FSAg)-activated dendritic cells were co-cultured with autologous CD4^+^ T cells for five days. Polarization of Th1, Th2, Th17, and regulatory T cell responses was analyzed by intracellular staining for IFN-γ, IL-4, IL-17A, and FoxP3, respectively. Representative dot-plots and mean ± SEM (*n* = 5 donors) values are presented. **c** Amount (pg/ml) of secretion of CD4^+^ T-cell cytokines IFN-γ, IL-4, and IL-17A in the dendritic cell–T cell co-cultures (mean ± SEM, *n* = 5 donors). **d**–**e** Inhibition of TLR4-signaling in dendritic cells abrogates T cell polarizing capacity of FSAg. Representative dot-plots for various CD4^+^ T cell subsets by intracellular staining for IFN-γ, IL-4, IL-17A, and FoxP3, and mean ± SEM (*n* = 5 donors) values are presented. *P < 0.05; **P < 0.01; ***P < 0.001; ****P < 0.0001; ns, not significant as analyzed by one-way ANOVA test. Abbreviation: Ctr, control

One of the main functions of “antigen-educated” dendritic cells is to promote T cell responses. Therefore, to characterize the CD4^+^ T cell responses and polarization elicited by microfilarial sheath antigen, we co-cultured “sheath antigen-educated” dendritic cells with autologous CD4^+^ T cells. CD4^+^ T cell response was measured by analyzing the different subsets of CD4^+^ T cells and cytokines secreted in the dendritic cell-T cell co-culture. We found that microfilarial sheath antigen elicited predominantly Th1 responses as demonstrated by enhanced IFN-γ-positive CD4^+^ T cells and high amount of IFN-γ secretion. In addition, microfilarial sheath antigen also mounted regulatory T cell responses (Fig. 6a–c). However, the frequency of Th2 and Th17 cells and the amount of secretion of corresponding T cell subset cytokines (IL-4 and IL-17A respectively) were marginal (Fig. 6a–c).

### *W. bancrofti* sheath antigen induced T cell responses are abrogated upon TLR4 inhibition

Since microfilarial sheath antigen-mediated dendritic cell maturation and activation were dependent on TLR4, we analyzed the effect of inhibition of TLR4 signaling in dendritic cells towards CD4^+^ T cell polarization. As demonstrated in Fig. 6d, e, microfilarial sheath antigen failed to induce any CD4^+^ T cell polarization when TLR4 signaling pathway was blocked in dendritic cells. The frequency of various effector CD4^+^ T cells (Th1, Th2, and Th17) and regulatory T cells induced by microfilarial sheath antigen when TLR4 was blocked in dendritic cells was similar to control conditions (Fig. 6d, e).

## Discussion

The mechanistic insights on the regulation of immune cell functions in filarial patients that shift host immunity from ‘protection’ to ‘pathology’ are still unclear. Majority of the studies on immunopathogenesis of lymphatic filariasis were focussed on *Brugia spp*., the least pathogenic human filarid and the only lymphatic filarial parasite that can be maintained in the laboratories^2,15^. This is contrast to *W. bancrofti* that cannot be maintained in the research laboratory. Therefore, research on *W. bancrofti* is relied on isolating the microfilariae from infected individuals. But this could be also considered as advantage since immunological studies by using these microfilariae represent what is actually happening in the affected population.

Recently, our studies have demonstrated that a sheath antigen FSAg of *W. bancrofti* acts as a ligand for TLR4 on macrophages and promotes proinflammatory responses by activating TLR4-NF-κB signaling cascade^13^. Although these data provided insight on the inflammatory responses elicited by *W. bancrofti*, the process by which these filariae promote T cell responses was not clear. As dendritic cells bridge innate and adaptive immunity due to their prime role in the polarization of diverse T cell responses, prompted us to examine the cross-talk between these professional antigen presenting cells and microfilarial sheath antigen.

Dendritic cell-filarid interactions lead to several changes in these innate cells and the outcome of these interactions appears to vary depending on the filarial species, antigens and density of filariae. Previous studies by using live *B. malayi* have shown that it induces certain level of dendritic cell death when used at high concentration^11^. At high density, *B. malayi* also inhibits capacity of dendritic cells to produce IL-12 and IL-10, and ability of dendritic cells to stimulate T cell cytokines^12^. But when used at low numbers, the cell death was marginal. In fact, asymptomatic *W. bancrofti*-infected individuals had enhanced number of circulating myeloid dendritic cells^16^. In this study, we show that sheath antigen of *W. bancrofti* induces maturation and activation of human dendritic cells with the secretion of various inflammatory cytokines that might recruit other immune cells. We did not observe substantial differences in the viability of microfilarial sheath antigen-treated dendritic cells vs control cells. Also, these dendritic cells promoted Th1 and regulatory T cell responses while Th2/Th17 polarization was marginal. Thus, our data suggest that *W. bancrofti* promotes inflammatory innate and adaptive T cell responses, and this phase possibly represents an early immune response to *W. bancrofti*. Of note, inflammatory responses induced from dendritic cell-filarid interactions are postulated to play key role in filarial immunopathogenesis^16–18^.

The concomitant regulatory T cell response induced by ‘sheath antigen-educated’ dendritic cells might help in the regulation of this acute inflammation and also the pathology induced by *W. bancrofti*^3^. However, in chronic lymphatic filariasis, like in the case of other chronic infections^19^, these regulatory T cells mediate suppression of immune responses and support persistence of parasite^20,21^. In fact, in vivo neutralization of regulatory T cells in mice improved *B. malayi* clearance^22^. It is not clear at this stage whether dendritic cell-mediated low Th2 response to microfilarial sheath antigen is specific to the antigen or due to regulatory T cell responses. Previous study using peripheral blood mononuclear cells from microfilaria-positive asymptomatic individuals has reported that depletion of regulatory T cells in in vitro cultures enhances Th2 response (IL-13) to *B. malayi* adult worm antigen^23^. Th1 response although enhanced in these conditions, it was not specific for the *B. malayi* adult worm antigen. This is in contrast to microfilarial sheath antigen that induced predominant Th1 responses. In the experimental settings where similar kind of T cell polarization was observed, inhibition of regulatory T cells lead to enhanced Th1 responses with no changes in the Th2 responses^24^. Therefore, it is likely that low Th2 response observed with FSAg is specific for the antigen rather than inhibition by regulatory T cells. Although in humans depletion of regulatory T cells in vitro lead to increased Th2 response to *B. malayi* adult worm antigen, regulatory T cell depletion in mice infected with of *B. malayi* did not lead to increased Th2 responses^22^.

Filariae could circumvent immune system and promote inflammation by expressing a variety of immune evasion products including molecules homologue of mammalian counterparts. *B. malayi* expresses homologues of human macrophage inhibitory factor (MIF), Bm-MIF-1 and Bm-MIF-2 that not only act as chemotactic agents for monocytes, but also induce IL-8, TNF-〈, and endogenous MIF^25^. The 70 kDa sheath antigen of *W. bancrofti* is a homologue of bestrophin^13^, a superfamily comprises of evolutionary related proteins that act as calcium-activated channels. Much is not known about the role of bestrophin in the regulation of immune responses. The fact that microfilarial sheath antigen promotes innate cell activation by binding through TLR4, should prompt further exploration of this superfamily in the regulation of immune responses.

**Fig. 7:**
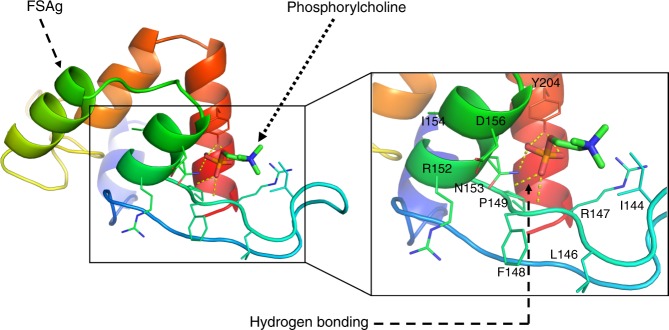
Molecular docking showing the interaction of microfilarial sheath antigen with phosphorylcholine. Crystal structure of phosphorylcholine was obtained from PubChem repository (PubChem ID: 135437). The 3D structure of microfilarial sheath antigen (FSAg) was obtained from our previously modelled structure^13^. The docking experiment was executed in silico employing Autodock Vina. Hydrogen bonds are depicted as yellow  dashed lines. The conformation presented here possesses the negative binding energy (−4.0 kcal/mol)

Filarial nematodes are known to contain phosphorylcholine-containing antigens^26–31^. However, to our knowledge, 70 KDa sheath antigen is the first report of phosphorylcholine-binding protein with a potent innate cell stimulatory capacity in the human filarid *W. bancrofti*. Molecular docking clearly revealed binding of microfilarial sheath antigen with phosphorylcholine. F148, N153, and Y204 amino acid residues of sheath antigen form hydrogen bonds with the phosphate group of phosphorylcholine (Fig. 7). This interaction is distinct to other phosphorylcholine-binding proteins from the cell wall of microbes like *Streptococcus pneumoniae* that attach to the phosphorylcholine of the cell wall of bacteria through conserved choline-binding domains^32–34^. These choline-binding proteins including hydrolases and adhesins have been reported to play a role in the virulence of *S. pneumoniae* by promoting bacterial colonization in nasopharynx^32–34^. Whether phosphorylcholine-binding properties of sheath antigen also contributes to the pathogenesis of filariasis need to be explored in the future.

Previously, excretory-secretory protein ES-62, one of the phosphorylcholine-containing antigens from the rodent filarial nematode *Acanthocheilonema viteae* was reported as a ligand for dendritic cell-TLR4^35–37^. Interaction between ES-62 and TLR4 on dendritic cells resulted in the disruption of TLR-mediated activation, pro-inflammatory responses as well as Th1 priming^36,37^. Such anti-inflammatory responses are postulated to play an important role in protecting the parasite from host immunity^36,37^. However, the identity of the pathogen-associated molecular pattern in human filarid that promotes inflammation and pathology is not yet known. Based on our earlier report^13^ and current findings, we suggest that sheath antigen, the *W. bancrofti* surface antigen is most likely the candidate that contributes to pathology in lymphatic filariasis by TLR4 signaling pathway and hence represents potential target for the immunotherapy.

## Methods

### Antibodies and reagents

For flow cytometry, the following fluorochrome-conjugated monoclonal antibodies were used. BD Biosciences: HLA-DR-APC (Clone: G46-6), CD86-FITC (Clone: FUN-1), CD80-PE (Clone: L307.4), CD54-APC (Clone: HA58), CD25-FITC (Clone: M-A251), CD127-BV421 (clone HIL-7R-M21), IFN-γ-FITC (Clone: 4S.B3), IL-4-PE (Clone: MP4-25D2); eBioscience: FoxP3-APC (Clone: 236A/E7), IL-17A-PE (Clone: Ebio64cap17); Beckman Coulter: CD40-PE (Clone: MAB89); Biolegend: CD4-PerCP (Clone: SK3). Cell viability was detected using the fixable viability dye eFluor 506 (eBioscience).

Antigen affinity-purified polyclonal anti-human TLR4 goat IgG was purchased from R&D systems. Cytokines (recombinant human granulocyte-macrophage colony-stimulating factor and IL-4), MicroBeads (CD14 and CD4) and cell purification units were obtained from Miltenyi Biotec. Protein-A agarose beads were from Cell Signalling Technology, and *E. coli* 055:B5 LPS and Polymyxin B-conjugated agarose beads were from Sigma-Aldrich. TLR4 signaling inhibitor CLI-095 and CpG ODN 2006 were procured from InvivoGen.

### Preparation of microfilarial sheath antigen

Microfilarial sheath antigen was prepared from the sheath of *W. bancrofti* microfilariae isolated from the blood of microfilaraemic patients^13^. Nocturnal blood sampling was conducted in filaria endemic villages of Birbhum district (24°35′ N and 88° 1′ 40″ E), West Bengal, India with the approval of Ethical Committee of Visva-Bharati University (1819/GO/Ere/S/15/CPCSEA) and Bolpur sub-divisional hospital, Bolpur, India. Informed consent was obtained from the subjects before blood collection. Heparinized blood was diluted (1:1) with phosphate buffered saline (pH 7.0) and centrifuged at 5000 rpm for 5 min at 25 °C. The pellet containing microfilariae was washed twice with phosphate-buffered saline and treated with antibiotic cocktail (100 U/ml penicillin, 100 μg/ml streptomycin and 10 μg/ml amphotericin B) for 6 h followed by polymyxin B (10 μg/ml) treatment for another 6 h. Parasites were retrieved by centrifugation (5000 rpm for 2 min), washed thrice with 50 mM phosphate-buffered saline (pH 7.4) and finally suspended in phosphate-buffered saline. Microfilarial surface layers (sheath) were isolated according to Klonisch et al.^38^ and lysate was prepared in ice-cold phosphate-buffered saline (50 mM, pH 7.4) containing protease inhibitors (Fermentus) by homogenization and ultrasonication (10 cycles each of 30 s at 20 KHz). The homogenate was centrifuged (15,000 rpm for 30 min at 4 °C) and supernatant was stored at −20 °C until further use.

Microfilarial sheath antigen was isolated through bioactivity^13^ and molecular docking-guided isolation procedure. In brief, the supernatant was centrifuged (15,000 rpm for 30 min at 4 °C) and supernatant was fractioned by ultrafiltration using 50 kDa molecular weight cut off membrane filters (Millipore). Fifty micrograms of total protein from the two fractions viz. >50 kDa and <50 kDa were separately added to RAW 264.7 macrophage cell line (1 × 10^6^). Greater than fifty kilodalton fraction was found to be active as it caused inflammatory responses in macrophages and the release of TNF-α. Therefore, >50 kDa fraction was subjected to gel filtration chromatographic separation on a sephadex G75 column (2 × 50 cm). As reported earlier^14^, gel filtration chromatogram revealed appearance of three protein rich peaks corresponding to microfilarial sheath antigen.

**Fig. 8:**
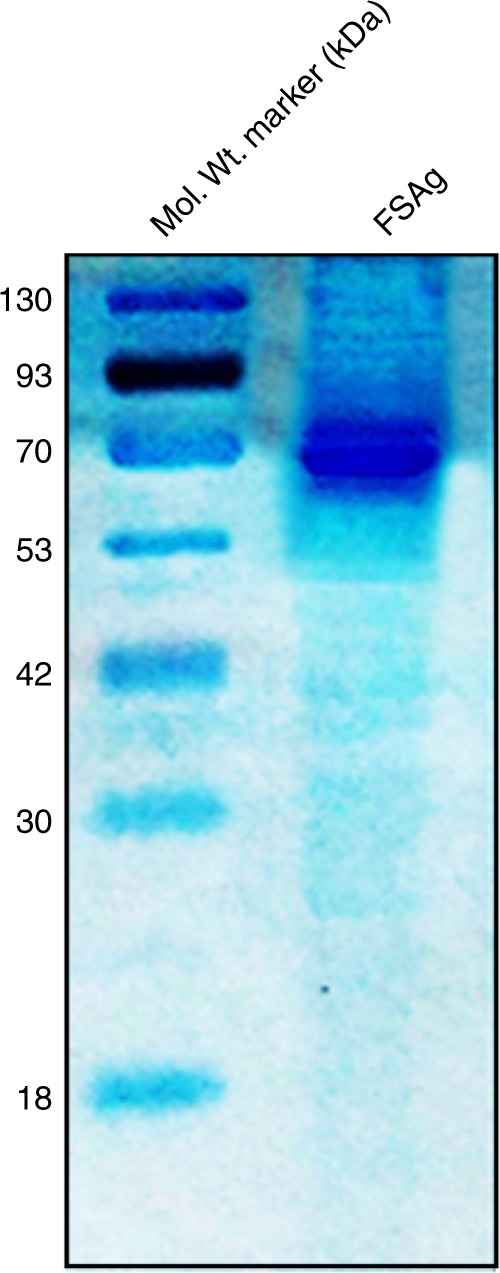
SDS-PAGE analysis of purified microfilarial sheath antigen (FSAg)

As sheath antigen was a newly identified transmembrane antigen of *W. bancrofti*, the purification of this antigen was challenging. In the beginning we opted for a trial and error strategy for the purification of microfilarial sheath antigen by using a number of columns including hydroxyapatite and C18. However, none of the matrix was effective in purifying the antigen. Based on the molecular docking studies, we ultimately selected phosphorylcholine as an affinity ligand for the purification of sheath antigen. For molecular docking, crystal structure of phosphorylcholine was obtained from PubChem repository (PubChem ID: 135437). The 3D structure of microfilarial sheath antigen was obtained from our previously modelled structure^13^. The docking experiment was executed in silico employing Autodock Vina. Molecular docking experiments revealed hydrogen bonds-mediated binding of microfilarial sheath antigen to phosphate group of phosphorylcholine. Therefore as detailed earlier^13^, further purification of sheath antigen was performed by using p-amino phenyl phosphorylcholine agarose (Thermo Scientific). Microfilarial sheath antigen was resolved in 10% SDS-PAGE and purified antigen was appeared as a single band (Fig. 8). The yield of sheath antigen was about 100 μg from nearly 10000 parasites.

Prior its use in cell stimulation experiments, microfilarial sheath antigen was treated with polymyxin B-conjugated agarose column (Sigma-Aldrich) following manufacturer’s guidelines. Five hundred micrograms of total antigen was loaded on the column, incubated for 6 h at 4 °C and the antigen was eluted with endotoxin-free phosphate-buffered saline. The endotoxin level in polymyxin-B-treated microfilarial sheath antigen (5 μg/ml) as determined by Pierce™ Chromogenic Endotoxin Quant Kit (ThermoFisher) was 0.46 EU/ml. Protein quantity was determined by NanoDrop™ 2000 spectrophotometer (Thermo Scientific).

### Generation of dendritic cells

Peripheral blood monocytes were isolated from buffy bags of healthy donors (Centre Necker-Cabanel, L'Établissement français du sang, Paris; INSERM-EFS ethical committee permission N°15/EFS/012, 18/EFS/033) by using CD14 magnetic beads and were differentiated to dendritic cells by culturing them for six days in complete medium (RPMI-10% fetal calf serum) in the presence of granulocyte-macrophage colony-stimulating factor (1000 IU/10^6^ cells) and IL-4 (500 IU/10^6^ cells)^39^.

### Culture of dendritic cells with microfilarial sheath antigen

The differentiated immature dendritic cells (0.5 × 10^6^/ml) were cultured in complete medium (RPMI-10% fetal calf serum) supplemented with granulocyte-macrophage colony-stimulating factor and IL-4 either alone or with sheath antigen or LPS (positive control; 100 ng/ml) or CpG ODN (positive control; 5 μM) for 24 h. In addition, as a control, dendritic cells were also stimulated with 50 pg/ml of LPS to prove that residual minute endotoxins in microfilarial sheath antigen have no capacity to stimulate dendritic cells.

Dendritic cells were processed for the surface staining of CD80, CD86, CD40, HLA-DR, and CD54, and acquired using flow cytometer (LSR II, BD Biosciences). The expression of various molecules was analyzed by BD FACS DIVA and FlowJo. The gating strategy for dendritic cells is provided in the Supplementary Fig. 1a

By enzyme-linked immunosorbent assay (ELISA Ready-SET-Go, eBioscience), the cell free supernatants were analyzed for the various dendritic cell cytokines viz. TNFα, IL-6, IL-1β, IL-10, IL-12p70, and IL-8.

For TLR4 signaling blocking experiments, dendritic cells were pre-incubated with CLI095 (3 µM) or 0.1% dimethyl sulfoxide before treating with sheath antigen (5 µg/0.5 × 10^6^ cells) for 24 h.

### Coculture of dendritic cells and CD4^+^ T cells

CD4^+^ T cells were isolated from the peripheral blood mononuclear cells using CD4 magnetic beads (Miltenyi Biotec) and subjected to autologous mixed lymphocyte reaction with control and microfilarial sheath antigen-educated dendritic cells at 20:1 ratio for five-days in serum-free X-VIVO medium. For analyzing T-cell polarization (Th1, Th2, Th17, and regulatory T cells), after five days culture, cells were activated with phorbol 12-myristate 13-acetate (50 ng/ml/0.5 × 10^6^ cells, Sigma-Aldrich) and ionomycin (500 ng/ml/0.5 × 10^6^ cells, Sigma-Aldrich) along with GolgiStop (BD Biosciences) for 4 h. Cells were processed and stained with fluorochrome-conjugated monoclonal antibodies corresponding to surface molecules (CD4, CD25, CD127). Cells were then fixed and permeabilized using Foxp3 Fixation/Permeabilization kit (eBioscience) and incubated with fluorochrome-conjugated monoclonal antibodies for intracellular (FoxP3, IFN-γ, IL-4, IL-17A) molecules followed by flow-cytometry^24^. The gating strategy for CD4^+^ T cells is provided in the Supplementary Fig. 1b.

The cell-free culture supernatants from dendritic cell-T cell co-cultures were analyzed by ELISA for T cell cytokines (IFN-γ, IL-4, IL-17A) (ELISA Ready-SET-Go, eBioscience).

### Binding of sheath antigen with TLR4 on dendritic cells

Anti-sheath antigen antibody was developed in BALB/c mice and purified from the mice sera^13^. For co-immunoprecipitation of TLR4 and microfilarial sheath antigen followed by immunoblotting^13^, dendritic cells (0.5 × 10^6^) after the treatment with sheath antigen (10 and 25 μg) were lysed in immunoprecipitation buffer (50 mM Tris-HCl [pH 7.4], 1% NP-40, 0.25% Na-deoxycholate, 150 mM NaCl, 1 mM ethylenediaminetetraacetic acid, 1 mM phenylmethylsulfonyl fluoride, 1 mg/ml each aprotinin, leupeptin, and pepstatin, 1 mM sodium orthovanadate, 1 mM sodium fluoride). Protein A agarose beads were incubated overnight at 4 °C with 10 μg of anti-TLR4 or anti-sheath antigen antibodies. The antibody-bound beads were washed thrice, and added to dendritic cell lysate containing 500 μg of total protein and incubated for 4 h at 4 °C. The beads were harvested by centrifugation (10000 × *g* for 2 min at 4 °C), washed with phosphate-buffered saline. The immunoprecipitated proteins were eluted by heating at 60 °C for 15 min in Laemmli sample buffer. Samples of equal protein content were resolved by 10% SDS-PAGE and electro-transferred on to polyvinyl difluoride membranes (Millipore). Membranes were blocked with 5% bovine serum albumin prepared in TBST (0.02 M Tris-HCl [pH 7.5], 0.15 M NaCl and 0.1% Tween 20) and subjected to reciprocal immunoblotting. Anti-TLR4 antibody mediated immunoprecipitated proteins were incubated with anti-sheath antigen antibody for overnight at 4 °C and anti- sheath antigen-driven immunoprecipitated proteins were immunoblotted with anti-TLR4 antibody. Membranes were washed with TBST and incubated with horseradish peroxidase-conjugated secondary antibody for 4 h at room temperature. Membranes were washed thrice with TBST and developed with ECL detection system (ThermoFisher). Full, uncropped blot is provided in the Supplementary Fig. 2.

Microfilarial sheath antigen was labelled with FITC by using FITC labeling kit (Calbiochem). Dendritic cells (0.25 × 10^6^ cells) were incubated with 5 μg of FITC-labelled sheath antigen at 37 °C for 2 h. In other experiments, dendritic cells were treated with anti-TLR4 antibody (2 μg/0.25 × 10^6^ cells) for 2 h prior to incubation with FITC-labelled sheath antigen (5 μg/0.25 × 10^6^ cells). Cells were washed thrice with phosphate-buffered saline and analyzed by confocal microscopy (Leica) at ×40 magnification.

### Statistical analysis

Statistical analyses were performed by one-way ANOVA with Tukey’s multiple comparison post-test or two-way Mann–Whitney *U* test and *P* < 0.05 was considered as significant.

### Reporting summary

Further information on experimental design is available in the Nature Research Reporting Summary linked to this article.

## Supplementary information

Description of Supplementary Data

Reporting Summary

Supplementary Information

Supplementary Data 1

## Data Availability

The authors declare that all data supporting the findings of this study are available within the paper and its supplementary information. Source data underlying the graphs presented in the main figures is available in Supplementary Data 1.
