# Supplementary material for: Wuchereria bancrofti filaria activates human dendritic cells and polarizes T helper 1 and regulatory T cells via toll-like receptor 4
Source: Commun Biol. 2019 May 7;2:169. doi: 10.1038/s42003-019-0392-8 (PMC6505026; doi:10.1038/s42003-019-0392-8)
Supplement: Supplementary file 1 — Description of Supplementary Data [file 42003_2019_392_MOESM1_ESM.docx]

**Description of Additional Supplementary Files**

**File Name**: Supplementary Data 1

**Description**: source data for all the graphs
