# Supplementary material for: Wuchereria bancrofti filaria activates human dendritic cells and polarizes T helper 1 and regulatory T cells via toll-like receptor 4
Source: Commun Biol. 2019 May 7;2:169. doi: 10.1038/s42003-019-0392-8 (PMC6505026; doi:10.1038/s42003-019-0392-8)
Supplement: Supplementary file 2 — Reporting Summary [file 42003_2019_392_MOESM2_ESM.pdf]

nature research

Corresponding author(s): Jagadeesh Bayry

Reporting Summary

Nature Research wishes to improve the reproducibility of the work that we publish. This form provides structure for consistency and transparency in reporting. For further information on Nature Research policies, see Authors & Referees and the Editorial Policy Checklist.

Statistical parameters

When statistical analyses are reported, confirm that the following items are present in the relevant location (e.g. figure legend, table legend, main text, or Methods section).

n/a	Confirmed	
☐	☒	The exact sample size (n) for each experimental group/condition, given as a discrete number and unit of measurement
☐	☒	An indication of whether measurements were taken from distinct samples or whether the same sample was measured repeatedly
☐	☒	The statistical test(s) used AND whether they are one- or two-sided<br>*Only common tests should be described solely by name; describe more complex techniques in the Methods section.*
☒	☐	A description of all covariates tested
☐	☒	A description of any assumptions or corrections, such as tests of normality and adjustment for multiple comparisons
☐	☒	A full description of the statistics including central tendency (e.g. means) or other basic estimates (e.g. regression coefficient) AND variation (e.g. standard deviation) or associated estimates of uncertainty (e.g. confidence intervals)
☐	☒	For null hypothesis testing, the test statistic (e.g. F, t, r) with confidence intervals, effect sizes, degrees of freedom and P value noted<br>*Give P values as exact values whenever suitable.*
☒	☐	For Bayesian analysis, information on the choice of priors and Markov chain Monte Carlo settings
☒	☐	For hierarchical and complex designs, identification of the appropriate level for tests and full reporting of outcomes
☒	☐	Estimates of effect sizes (e.g. Cohen's d, Pearson's r), indicating how they were calculated
☐	☒	Clearly defined error bars<br>*State explicitly what error bars represent (e.g. SD, SE, CI)*

Our web collection on statistics for biologists may be useful.

Software and code

Policy information about availability of computer code

Data collection	BD FACS DIVA for flow cytometry
Data analysis	GraphPad Prism, BD FACS DIVA, FlowJo, Microoift Excel, Autodock Vina

For manuscripts utilizing custom algorithms or software that are central to the research but not yet described in published literature, software must be made available to editors/reviewers upon request. We strongly encourage code deposition in a community repository (e.g. GitHub). See the Nature Research guidelines for submitting code & software for further information.

Data

Policy information about availability of data

All manuscripts must include a data availability statement. This statement should provide the following information, where applicable:

- Accession codes, unique identifiers, or web links for publicly available datasets
- A list of figures that have associated raw data
- A description of any restrictions on data availability

All data are included in this article and figures. No restrictions on the data availibility

April 2018

Field-specific reporting

Please select the best fit for your research. If you are not sure, read the appropriate sections before making your selection.

☒ Life sciences ☐ Behavioural & social sciences ☐ Ecological, evolutionary & environmental sciences

For a reference copy of the document with all sections, see nature.com/authors/policies/ReportingSummary-flat.pdf

Life sciences study design

All studies must disclose on these points even when the disclosure is negative.

Sample size	Sample sizes of five per group were chosen as this would allow the detection of a 25% difference in the mean between experimental and control groups with a probability of greater than 95% ($p < 0.05$). In cases where no significant differences were observed during the experiments, sample size was limited to three. Acquisition of data from multiple independent donors allowed us to control inter-individual variations.
Data exclusions	Only data from the experiments with: technical errors (such as unsuccessful stimulation or staining) or unsatisfactory quality of buffy bags and cells were excluded from the analysis.
Replication	Replication is ensured by the use of several independent donors
Randomization	There was no intervention. Hence, randomization was not used.
Blinding	Blinding was not used as there was no intervention in the project

Reporting for specific materials, systems and methods

Materials & experimental systems

n/a	Involved in the study	
☐	☒	Unique biological materials
☐	☒	Antibodies
☒	☐	Eukaryotic cell lines
☒	☐	Palaeontology
☒	☐	Animals and other organisms
☐	☒	Human research participants

Methods

n/a	Involved in the study	
☒	☐	ChIP-seq
☐	☒	Flow cytometry
☒	☐	MRI-based neuroimaging

Unique biological materials

Policy information about availability of materials

Obtaining unique materials	No restrictions

Antibodies

Antibodies used	For flow cytometry, the following antibodies were used. BD Biosciences: HLA-DR-APC (Clone: G46-6), CD86-FITC (Clone: FUN-1), CD80-PE (Clone: L307.4), CD54-APC (Clone: HA58), CD25-FITC (Clone: M-A251), CD127-BV421 (clone HIL-7R-M21), IFN-γ-FITC (Clone: 4S.B3), IL-4-PE (Clone: MP4-25D2); eBioscience: FoxP3-APC (Clone: 236A/E7), IL-17A-PE (Clone: Ebio64cap17); Beckman Coulter: CD40-PE (Clone: IMAB89); Biolegend: CD4-PerCP (Clone: SK3). Cell viability was detected using the fixable viability dye eFluor 506 (eBioscience).<br>R&D Systems: Antigen affinity-purified polyclonal anti-human TLR4 goat IgG<br>Anti-FSAg antibody was generated and validated as previously detailed (Mukherjee et al. J Infect Dis 2017)
Validation	Flow-cytometry antibodies were validated by manufacturers. Also they were validated in the lab for previous reports (For example: Maddur et al Nat Commun 2014; Stephen-Victor et al. J Infect Dis 2017; Sharma et al. Science Immunol 2018). Anti-FSAg antibody has been previously validated (Mukherjee et al. J Infect Dis 2017)

Human research participants

Policy information about studies involving human research participants

Population characteristics	FSAg was prepared from the sheath of W. bancrofti microfilariae isolated from the blood of microfilaraemic patients at Birbhum district (24°35′ N and 88° 1′ 40″ E), West Bengal, India. Written consent was obtained before blood collection. Buffy bags of healthy donors of a non-endemic country (France) were purchased from the Centre Necker-Cabanel, EFS, Paris. The identity of the donors are blinded at purchase
Recruitment	Microfilaraemic patients were from Birbhum district (24°35′ N and 88° 1′ 40″ E), West Bengal, India. Buffy bags of healthy donors were purchased from the Centre Necker-Cabanel, EFS, Paris.

Flow Cytometry

Plots

Confirm that:

- [x] The axis labels state the marker and fluorochrome used (e.g. CD4-FITC).
- [x] The axis scales are clearly visible. Include numbers along axes only for bottom left plot of group (a 'group' is an analysis of identical markers).
- [ ] All plots are contour plots with outliers or pseudocolor plots.
- [x] A numerical value for number of cells or percentage (with statistics) is provided.

Methodology

Sample preparation	Peripheral blood monocytes were obtained from buffy bags of healthy donors and differentiated to DCs as previously detailed.15 Immature DCs (0.5x106/ml) were cultured in RPMI-10% FCS the presence of GM-CSF and IL-4 alone or with FSAg or lipopolysacharide (LPS, positive control; 100 ng/ml) or CpG ODN (positive control; 5 µM) for 24 hours. In addition, as a control, DCs were also stimulated with 50 pg/ml of LPS to prove that residual minute endotoxins in FSAg have no capacity to stimulate DCs. Surface staining was performed with fluorescence-conjugated MAbs to CD80, CD86, HLA-DR, CD40 and CD54 by standard procedure.<br>CD4+ T cells were isolated from the PBMCs using CD4 magnetic beads (Miltenyi Biotec) and subjected to autologous MLR with control and FSAg-educated DCs at 20:1 ratio for five-days in serum-free X-VIVO medium. CD4+ T cells were washed, plated in 24 well plate with RPMI 1640-10%FCS medium and activated with PMA (50 ng/ml/0.5 million cells) and ionomycin (500 ng/ml/0.5 million cells), along with GolgiStop for 4 h. Surface staining was performed with fluorescence-conjugated MAbs to CD4, CD127 and CD25. Cells were then fixed, permeabilized using intracellular staining kit (eBioscience), and incubated at room temperature with fluorescence-conjugated MAbs to FoxP3, IFN-γ, IL-4, and IL-17A.<br>For flow cytometry, the following antibodies were used. BD Biosciences: HLA-DR-APC (Clone: G46-6), CD86-FITC (Clone: FUN-1), CD80-PE (Clone: L307.4), CD54-APC (Clone: HA58), CD25-FITC (Clone: M-A251), CD127-BV421 (clone HIL-7R-M21), IFN-γ-FITC (Clone: 4S.B3), IL-4-PE (Clone: MP4-25D2); eBioscience: FoxP3-APC (Clone: 236A/E7), IL-17A-PE (Clone: Ebio64cap17); Beckman Coulter: CD40-PE (Clone: IMAB89); Biolegend: CD4-PerCP (Clone: SK3). Cell viability was detected using the fixable viability dye eFluor 506 (eBioscience).
Instrument	LSR II (BD Biosciences)
Software	FACSDiva and FlowJo
Cell population abundance	MicroBeads from Miltenyi Biotec (CD14 and CD4) were used for isolating monocytes and CD4 T cells from PBMC.<br>As pure dendritic cell population was used, cells were directly analyzed on live cells. For CD4 T cell response, CD4 cells in DC-CD4 T cell co-culture were first gated on live cell population and were then analyzed for various T cell subsets.
Gating strategy	Initial gating using FSC/SSC plot to select cells and exclude debris, followed by selection of viable cells using fixable viability dye eFluor 506.<br>As pure dendritic cell population was used, surface markers were analyzed on live cells. For CD4 T cell response, CD4 cells in DC-CD4+ T cell co-culture were gated on live cell population and were assessed for various T cell subsets.

- [x] Tick this box to confirm that a figure exemplifying the gating strategy is provided in the Supplementary Information.
