# Supplementary material for: Wuchereria bancrofti filaria activates human dendritic cells and polarizes T helper 1 and regulatory T cells via toll-like receptor 4
Source: Commun Biol. 2019 May 7;2:169. doi: 10.1038/s42003-019-0392-8 (PMC6505026; doi:10.1038/s42003-019-0392-8)
Supplement: Supplementary file 3 — Supplementary Information [file 42003_2019_392_MOESM3_ESM.pdf]

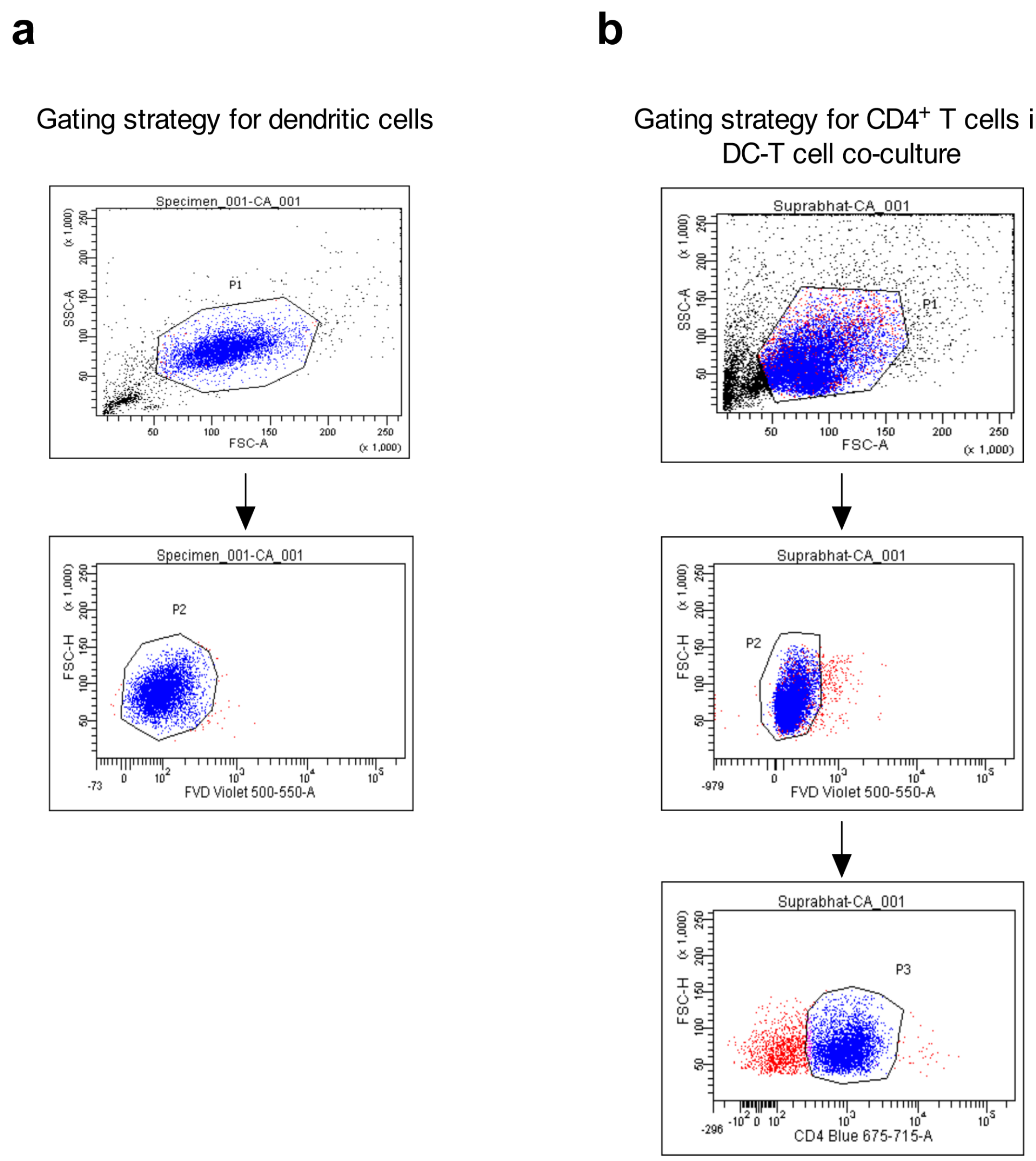

Supplementary Figure 1. The flow cytometer gating strategy for **(a)** dendritic cells (DCs) and **(b)** CD4$^+$ T cells.

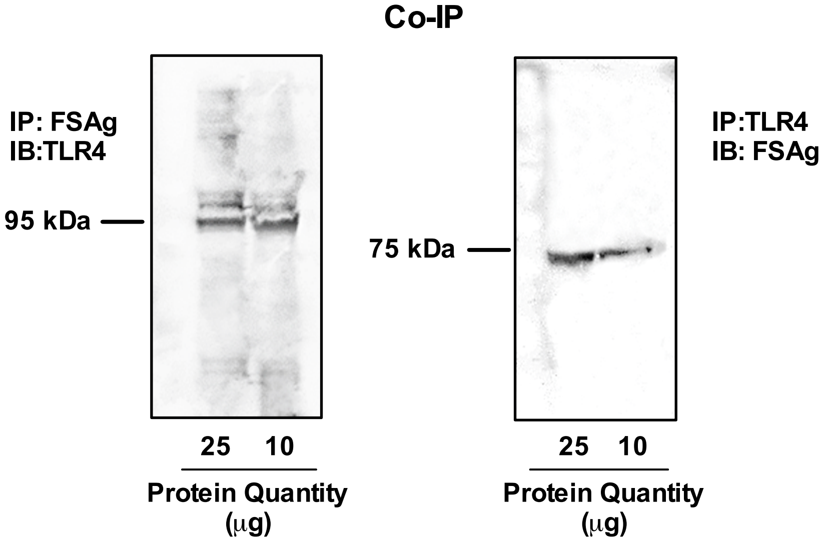

Supplementary Figure 2. Full, uncropped blot of co-immunoprecipitation of microfilarial sheath antigen (FSAg)–TLR4 complex followed by reciprocal immunoblotting using anti-FSAg or anti-TLR4 antibodies.
